# A novel fusion protein candidate for the serodiagnosis of *Mycoplasma agalactiae* infection

**DOI:** 10.1186/s12917-022-03558-0

**Published:** 2022-12-29

**Authors:** Malihe Akbarzadeh-Niaki, Abdollah Derakhshandeh, Nasrin Kazemipour, Farhid Hemmatzadeh

**Affiliations:** 1 Department of Pathobiology, Biotechnology Section, School of Veterinary Medicine, Shiraz University, Shiraz, Iran; 2 Department of Pathobiology, School of Veterinary Medicine, Shiraz University, Shiraz, Iran; 3 Department of Basic Sciences, School of Veterinary Medicine, Shiraz University, Shiraz, Iran; 4 School of Animal and Veterinary Sciences, The University of Adelaide, Adelaide, SA Australia

**Keywords:** *Mycoplasma agalactiae*, Fusion protein, Immunization, Serodiagnostic assay

## Abstract

**Background:**

The aim of current study was to construct, express, purify and immunogenicity evaluate of a novel recombinant fusion protein including Pyruvate dehydrogenase beta subunit (PDHB) and high antigenic region of lipoprotein P80 of *Mycoplasma agalactiae*. Using bioinformatics tools, antigenicity and physiochemical properties of fused protein were assessed.

**Material and methods:**

The recombinant fusion protein of GST-PDHB-P80 were expressed in pGEX4T-1 and purified then verified by Western blot assay. The purified protein was successfully used for immunization of mice. 30 female BALB/c mice were divided into three groups (10 mice per each group) injected with GST-PDHB-P80, inactivated bacteria vaccine and PBS as negative control, separately.

**Results:**

Western blot analysis confirmed the interaction between the immunized mice serum and the blotted recombinant protein GST-PDHB-P80, demonstrating the immunogenicity of this protein. Moreover, the sera of vaccinated mice with inactivated bacteria vaccine, containing whole cell proteins, detected the recombinant protein GST-PDHB-P80 confirming the antigenicity of PDHB-P80. Negative control displayed no reactivity with GST-PDHB-P80.

**Conclusion:**

We proposed a novel designed chimeric protein of *Mycoplasma agalactiae* as a potential marker for serodiagnostic assays but still further field research is required.

**Supplementary Information:**

The online version contains supplementary material available at 10.1186/s12917-022-03558-0.

## Background

Contagious agalactia (CA) is a multifactorial syndrome affecting small ruminant and classically caused by *Mycoplasma agalactiae* [1]. The disease inflicts a variety of clinical signs including arthritis, keratoconjunctivitis and mastitis, leading decline or suppression of milk production and increase the mortality rate, which put CA into the list of economically notifiable diseases announced by World Organization for Animal Health (OIE) [2–5]. Other investigations revealed that the agent has the ability to exist in asymptomatic carriers even several months after early phases of the infection, resulting the promotion of chronic state and silent spread of disease [6–9]. Thus, the establishment of a proper diagnostic assay is crucial to implement an effective program for eradication of the disease. For several years, great effort has been devoted to the study of recombinant proteins that persuaded the researchers to focus on immunogenic proteins of *Mycoplasma agalactiae* such as P30, P40, P55, P48, MAG_1560, MAG_6130 and P80 as potential diagnostic markers [10–16]. As Tola et al. reported in 1997, the integral membrane lipoprotein P80 was expressed during the entire phases of the infection conserved among *Mycoplasma agalactiae* strains and has been observed at the first signs of the disease [17]. An internal fragment of P80 was expressed, purified and successfully reacted with lamb serum in Tola's study and was applied in developing a recombinant ELISA assay by Fusco et al. for diagnosis of CA [13, 14]. Data from these studies have suggested this antigenic protein as a potential marker for developing a serodiagnostic assay. Pyruvate dehydrogenase beta subunit (PDHB) is a main metabolic protein, which links the glycolytic pathway to the tricarboxylic (TCA) cycle. Although the major role of this phosphoprotein is played in the cytoplasm, several studies have revealed the localization of PDHB on the cell surface of Mycoplasma species to facilitate the pathogen adhesion to the host cell. *pdhb* disruption in *Mycoplasma agalactiae* mutants indicated an adverse effect on pathogen capacity to invade HeLa cells, demonstrating the involvement of PDHB in pathogen interactions with host cell [18–20]. Moreover, the immune reactivity of PDHB was reported in some *Mycoplasma* species such as *Mycoplasma capricolum subsp. capripneumoniae*, *Mycoplasma hyopneumoniae, Mycoplasma bovis* and *Mycoplasma gallisepticum* [19, 21–23]. Sun et al. (2014) applied recombinant PDHB of *Mycoplasma bovis* as coating antigen in ELISA and demonstrated the potential of this protein for detection of infection [19]. According to this research background, P80 and PDHB proteins were selected to construct an effective serodiagnostic marker.

In our previous work, we presented a newly designed recombinant fusion protein consisting of the full-length of PDHB and the antigenic region of P80 protein of *Mycoplasma agalactiae* fused by human IgG_4_ middle hinge linker. The proposed fusion protein expressed as MBP-PDHB-P80 protein via the expression vector pMAL-p5X, and suggested as an appropriate candidate for immunogenicity evaluation, but purifying of MBP-PDHB-P80 was not succeed [24]. This study was aimed to express and purify of PDHB-P80 via pGEX-4-T1 vector expressing PDHB-P80 recombinant protein fused to glutathione S-transferase tag protein (GST-PDHB-P80) and determining the immunogenicity of the purified protein.

## Results

### Bioinformatics’ analysis

#### Secondary structure prediction

**Fig. 1:**
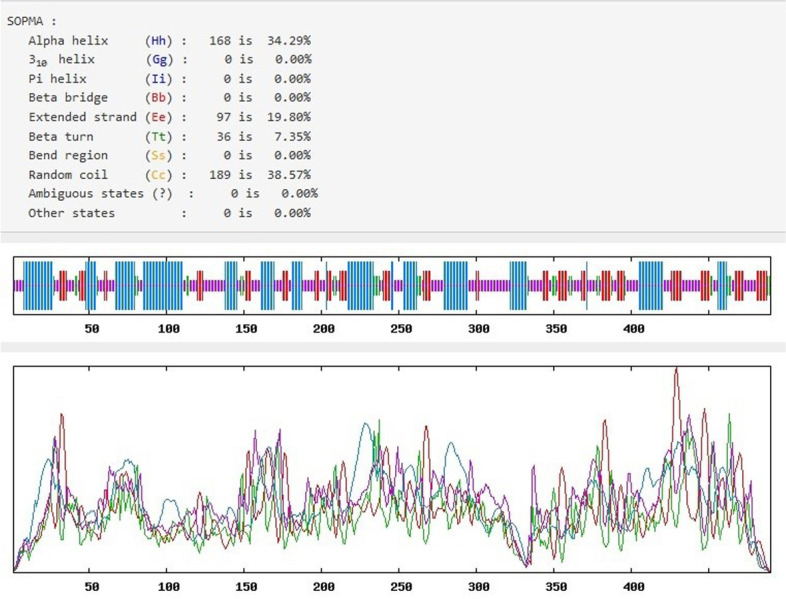
Secondary structure of PDHB-P80 estimated by SOPMA online server

Using SOPMA online server, we estimated the conformational states of secondary structure of PDHB-P80. According to the results, the ratio of α helices, extended strands, β turns and extended strands accounted for 34.29, 19.8, 7.35 and 38.57% of the secondary structure, respectively (Fig. 1).

#### Solubility

**Fig. 2:**
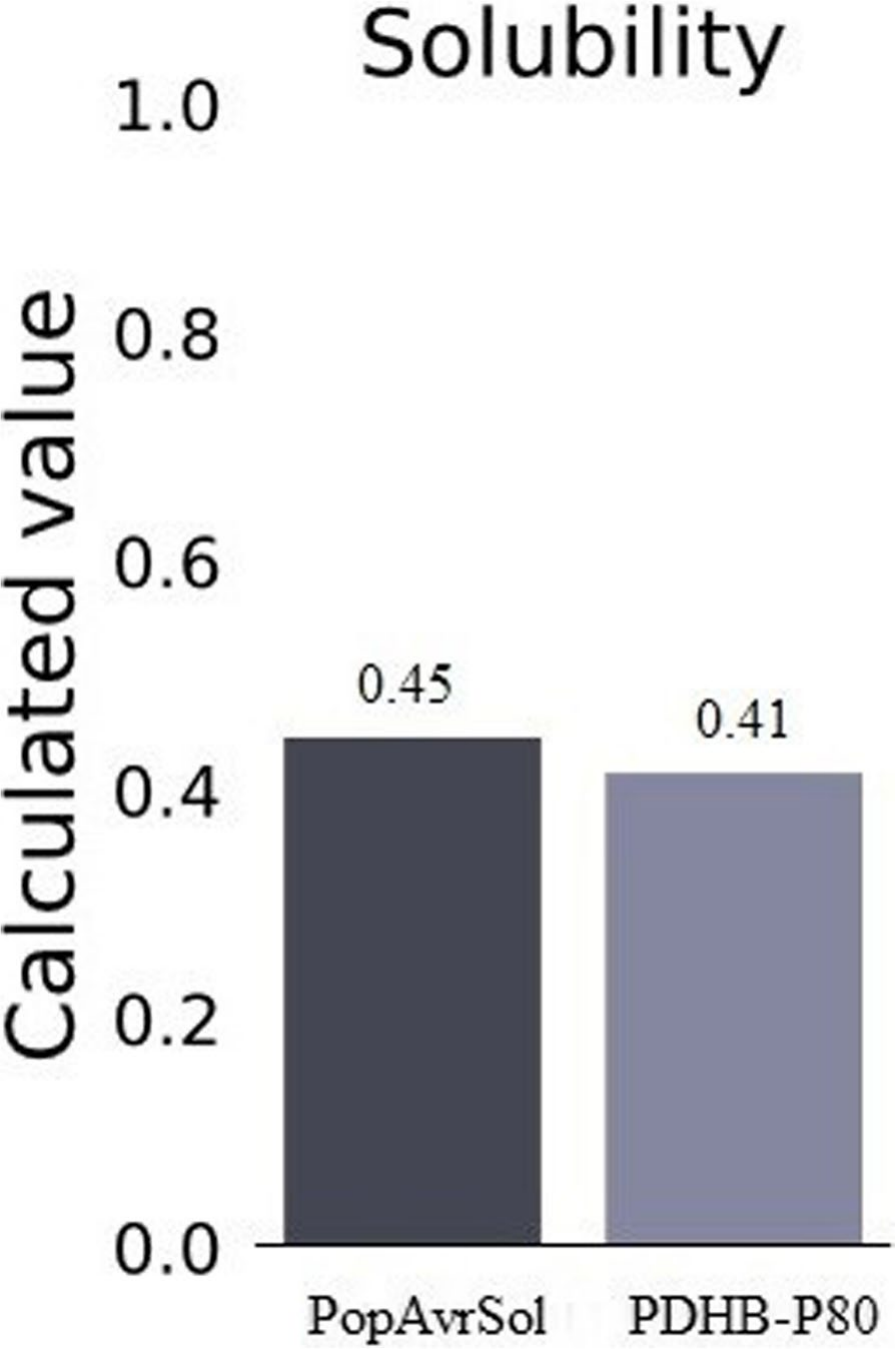
The solubility prediction of PDHB-P80 fusion protein by Pro-Sol online server

We applied Pro-Sol online server to determine the solubility of PDHB-P80 fusion protein. As is clear from Fig. 2, the comparison of PDHB-P80 solubility with the average soluble *E. coli* proteins indicated an appropriate solubility value around 0.41.

#### Hydrophobic regions

**Fig. 3:**
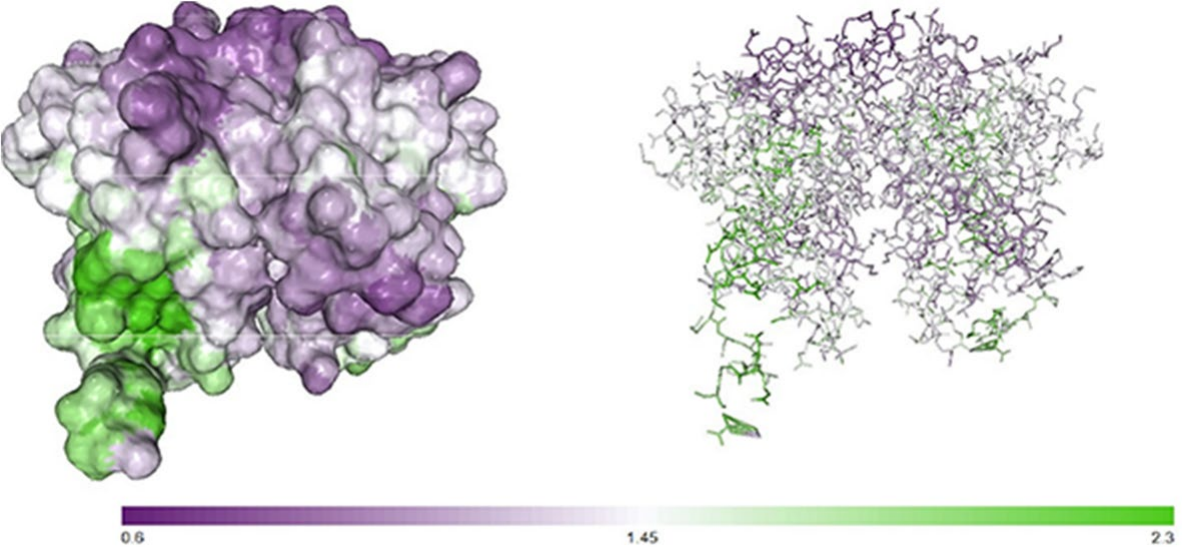
The prediction of the ratio of polar to non-polar regions of PDHB-P80 by protein-sol patches software. Polar region shown by purple and non-polar region by green

Results obtained from the prediction of the ratio of polar to non-polar regions of PDHB-P80 by protein-sol patches software indicated the dominant polarity of this fusion protein shown by purple in Fig. 3.

#### Prediction of the B-cell epitopes for the PDHB-P80 protein

**Fig. 4:**
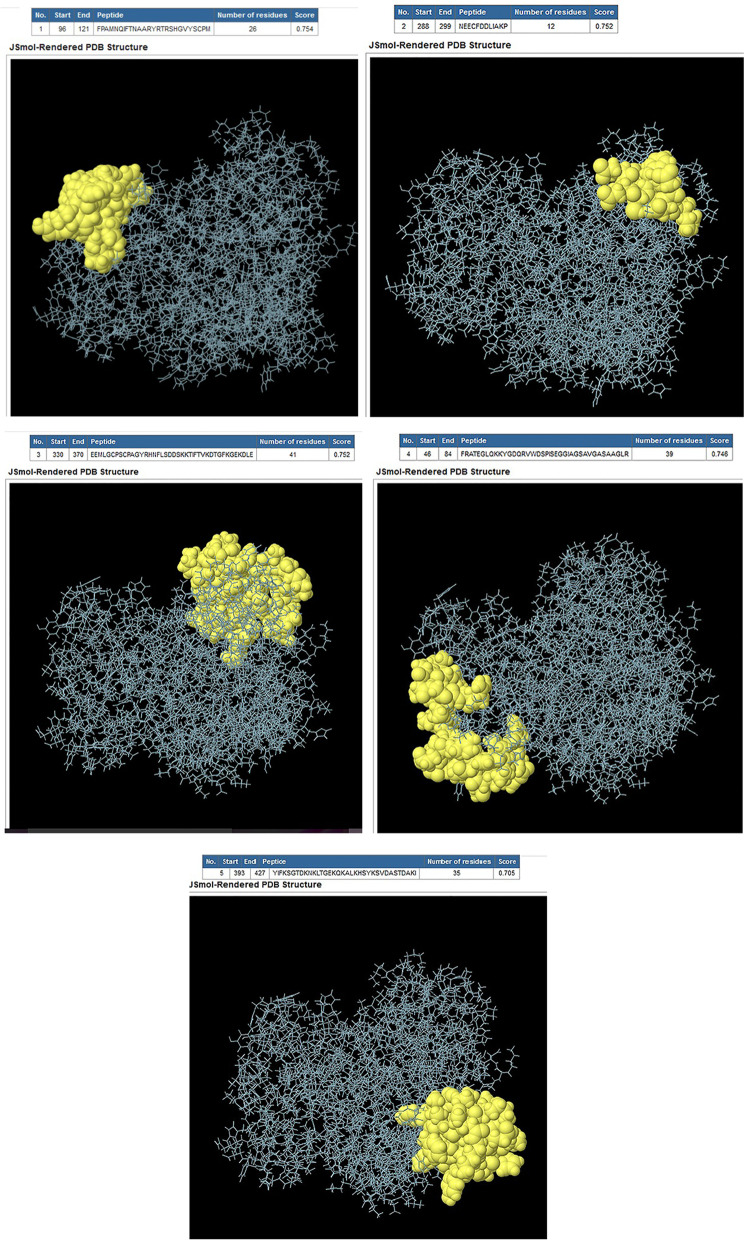
Estimation of three-dimensional structure of B-cell epitopes of the PDHB-P80 using ElliPro tool. Epitopes are shown as yellow spheres

We predicted the B-cell epitopes using ElliPro tool and considered the highest score epitopes as appropriate epitopes (Table 1). The three-dimension of the epitope residues were shown as yellow spheres in Fig. 4, the rest of the protein is in violet. The separated bioinformatics analyses for PDHB and P80 were performed and shown in supplementary file as Figs. 1S through 5S.

**Table 1:** Conformational B-cell epitopes predicted using ElliPro tool

Epitope location	Peptide sequence	Score
96–121	FPAMNQIFTNAARYRTRSHGVYSCPM	0.754
288–299	NEECFDDLIAKP	0.752
330–370	EEMLGCPSCPAGYRHNFLSDDSKKTIFTVKDTGFKGEKDLE	0.752
46–84	FRATEGLQKKYGDQRVWDSPISEGGIAGSAVGASAAGLR	0.746
393–427	YIFKSGTDKNKLTGEKQKALKHSYKSVDASTDAKI	0.705

**Fig. 5:**
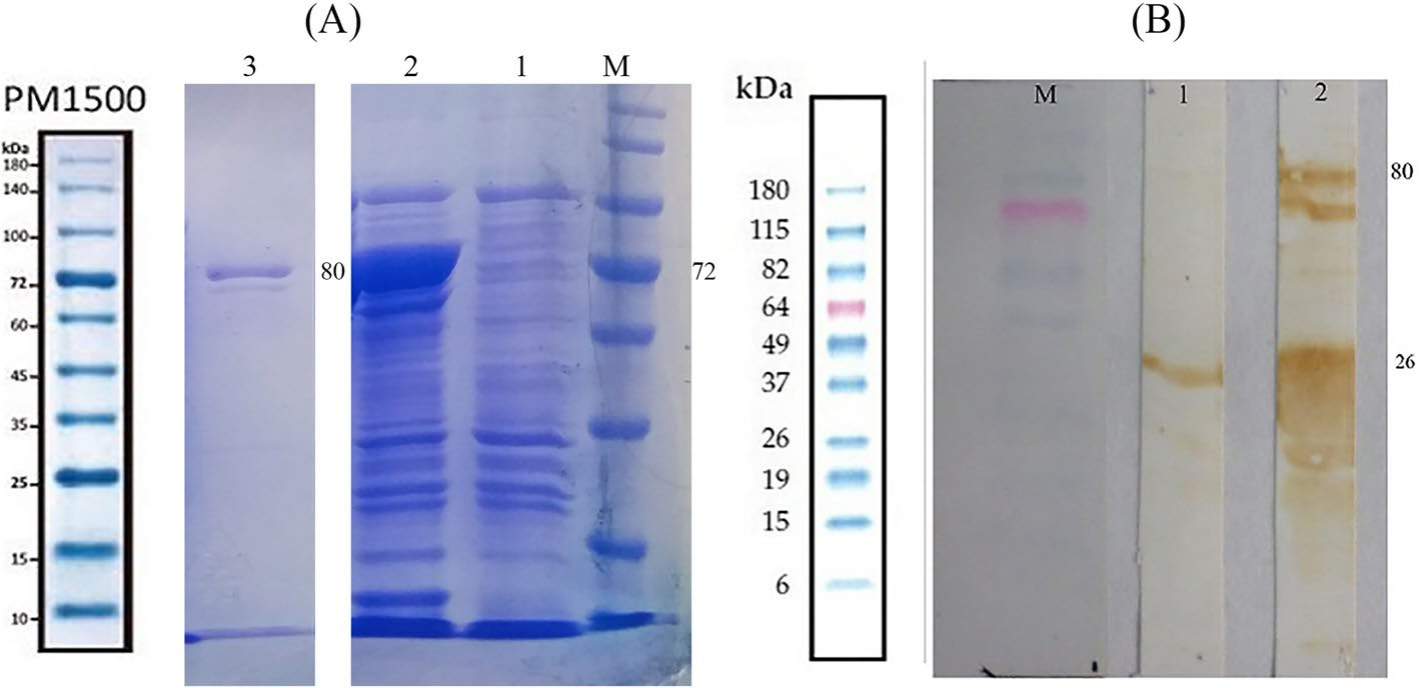
**A** SDS-PAGE analysis, M) protein marker PM1500, 1) non-induced cells, 2) induced cells expressing GST-PDHB-P80 protein on size 80 kDa, 3) purified GST-PDHB-P80 protein (**B**) Western blotting of the recombinant fusion protein PDHB-P80, M) protein marker BenchMark™ Pre-Stained 1) purified GST protein as control, 2) purified GST-PDHB-P80 protein

### Experiments

#### Cloning procedure

The cloning of the synthetic fusion gene *pdhb-p80* into the expression vector pGEX-4T1 was successfully performed and colony PCR, double digestion analysis and Sanger sequencing on the obtained recombinant colonies, confirmed the accurate insertion of *pdhb-p80* into the vector.

#### Protein expression and purification

A concentration of one mM Isopropyl β- d-1-thiogalactopyranoside (IPTG) was used to induce the expression of the recombinant GST-PDHB-P80 and maximum expression level was detected at four hours after induction. GST tag protein expressed by expression vector pGEX-4T1 has shown to be effective in improvement of solubility and yield of protein [25]. SDS-PAGE analysis result showed the expected band around 80 kDa in induced cells, indicating the proper expression of GST-PDHB-P80, which was not existed in non-induced cells (Fig. 5A). Furthermore, Western Blot analysis using Anti-GST visualized protein band around 80 kDa, confirming the presence of the purified GST-PDHB-P80 (Fig. 5B).

#### Immunization

**Fig. 6:**
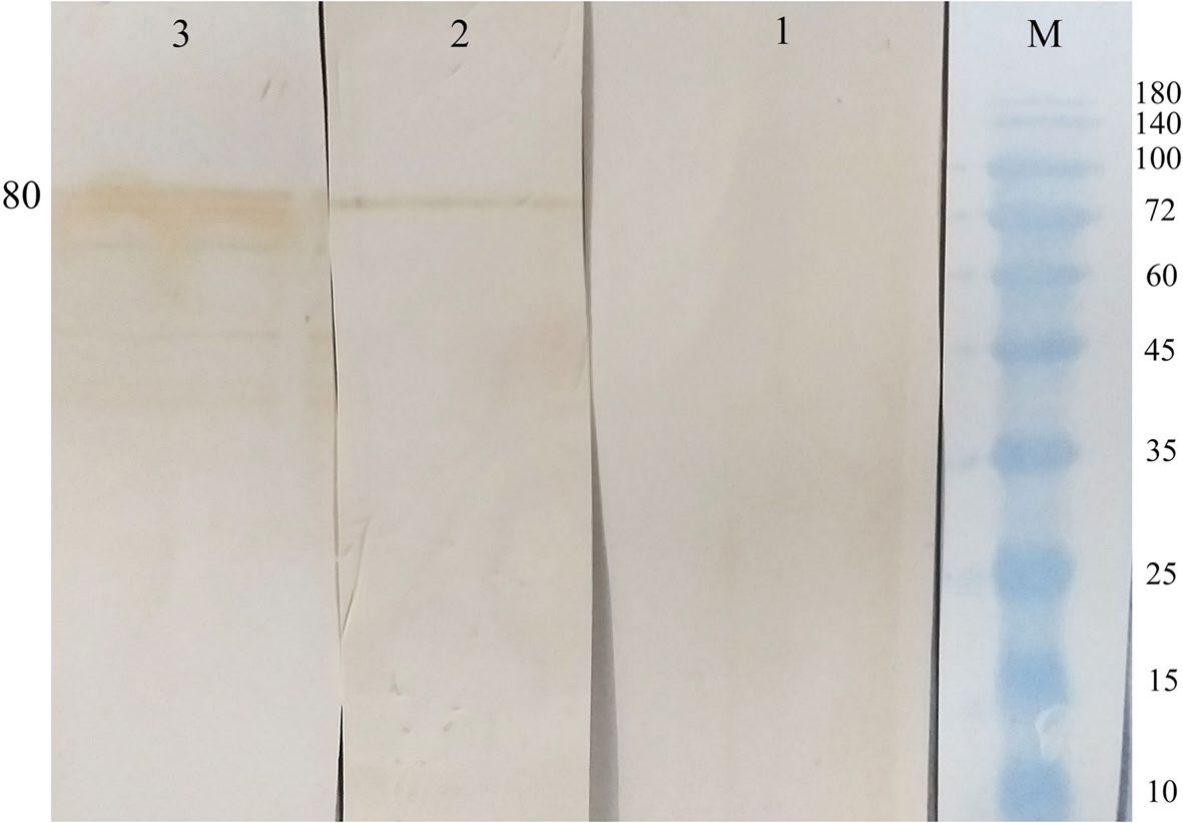
Analysis of PDHB-P80 immunization using Western Blot assay. M) Protein marker PM1500, 1) sera of mice treated with PBS as control, 2) sera of mice vaccinated with inactivated bacteria vaccine, 3) sera of mice immunized mice with GST-PDHB-P80

As mentioned earlier, we immunized BALB/c mice with purified recombinant protein GST-PDHB-P80 to examine the immune reactivity of our constructed recombinant protein. As shown in Fig. 6, Western blot analysis using Goat Anti-Mouse IgG (H L)-HRP antibody (Bio-Rad, U.S.A) indicated that the sera raised from immunized mice with GST-PDHB-P80 successfully detected the blotted GST-PDHB-P80, confirming the immunogenicity of this recombinant protein. Furthermore, we considered an alternative group vaccinated with inactivated bacteria vaccine, containing whole cell proteins to validate the immune reactivity of PDHB-P80. As may be seen in Fig. 6, the vaccinated mice sera were successfully reacted with the blotted GST-PDHB-P80, which has confirming the immunogenicity of PDHB-P80. No immune reactivity was observed from the sera of mice treated with PBS in negative control.

## Discussion

*Mycoplasma agalactiae* is a menace to the worldwide dairy industry causing severe economic losses annually [4, 5]. Prior researches have suggested different antigenic proteins for an early, effective and specific detection of infected animals including diseased and asymptomatic carriers [10–16]. Our paper presents an innovative constructed fusion protein composed of full length of PDHB and antigenic region of P80 as serodiagnostic marker for the detection of *Mycoplasma agalactiae* infection.

There is a rapidly growing literature on PDHB in various species of Mycoplasma, which demonstrate potent antigenicity of this phosphoprotein. Dallo et al. (2002) expressed and purified PDHB and indicated the immunogenicity of this protein by immunoblots of rabbit anti-rPDHB sera against *Mycoplasma pneumonia* total proteins [23]. Pinto et al. (2007) identified five highly antigenic proteins including PDHB by two-dimensional gel electrophoresis and immunoblots using the pig anti-*Mycoplasma hyopneumoniae* serum [21]. Zhao et al. (2012) detected nine immunogenic proteins including PDHB by two-dimensional gel electrophoresis of whole cell preparation and immunoblots using the serum of infected goat [22]. Sun et al. (2014) reported PDHB along with 19 highly immunogenic proteins of *Mycoplasma bovis* using 2-dimensional gel electrophoresis and immunoblots of the whole cell proteins with antisera from naturally infected cattle [19]. Qi et al. (2018) expressed and purified PDHB and demonstrated its immunogenicity by ELISA using immunized mice sera and Western blot using *Mycoplasma gallisepticum* infected chicken serum [26]. Regarding the data gathered in these studies, our research group has tended to focus on PDHB in *Mycoplasma agalactiae* for the first time.

P80 is an integral outer membrane protein conserved among *Mycoplasma agalactiae* strains. The data yielded by Tola' research illustrated that the expression of P80 entire protein could arrest the growth of *E. coli*. Thus, they successfully expressed a 37-kDa fragment of this lipoprotein that strongly reacted with the anti-rP80 lamb serum [13]. Fusco et al. (2007) applied this 37-kDa fragment along with P55 to stablish a recombinant ELISA for detection of CA [14]. In this paper, we employed the antigenic region of the fragment expressed by Tola linked to PDHB to strengthen the antigenicity of our proposed marker. Both proteins have been demonstrated to be expressed during the entire period disease. This stability is considered as a privilege for selecting a diagnostic marker [13, 17, 19]. Furthermore, host specificity is a main factor affecting serodiagnostic potency of a marker. The data obtained from protein BLAST of P80 amino acid sequence showed this protein is completely specific in *Mycoplasma agalactiae*. Sun et al. (2014) detected the cross reactivity of PDHB between *Mycoplasma bovis* and *Mycoplasma agalactiae*, which is not surprising, as these two species display 99.8% homology in 16S rRNA. Regarding different hosts of these two species, they considered this cross-reaction as a negligible issue for serodiagnosis of *Mycoplasma bovis* infection [19]. Supporting Sun et al., we applied PDHB to construct our recombinant fusion protein PDHB-P80.

Since the antigenic property of a protein is mainly affected by its secondary structure via determining the formation and distribution of epitopes, we predicted the proportion of different secondary structures α helices, extended strands, β turns and extended strands of PDHB-P80. The α helices and extended strands which are found inside the protein and maintain structural stability, accounted for 34.29 and 19.8% of the protein respectively. The random coils and β turns, which are located on the exposed area of the protein and represent the potential epitope regions accounted for 38.57 and 7.35% of PDHB-P80 protein respectively. These data suggested that PDHB-P80 protein has a stable structure and appropriate antigenicity property. Similarly, using SOPMA server, Forouharmehr, and Nassiry (2015) estimated the secondary structure of P40 protein of *Mycoplasma agalactiae* and demonstrated that the random coils and β turns have the higher possibility of forming epitopes [27, 28].

Prior studies have demonstrated the relationship between the higher solubility and hydrophilicity of a protein and the higher probability of epitopes formation and strong antigenicity [29]. Our analysis indicated a proper solubility and polarity of PDHB-P80 protein, which increase the possibility of epitope formation. As we successfully estimated B-cell epitopes distributed in three-dimensional structure of PDHB-P80 protein with desirable scores.

Using pGEX 4 T-1, the expression of GST-PDHB-P80 fusion protein was induced and we have obtained the expected 80 kDa protein band on SDS-PAGE and Western blot analysis. Along with the target protein, two other 70 and 26 kDa protein bands were observed, which are probably owing to the protein degradation or premature termination in ribosomes [30]. Regarding the specific reaction of these proteins against Anti-GST-HRP antibody, we can consider them as truncated forms of GST-PDHB-P80 protein. Similar to our study, other studies on the expression of different recombinant GST fusion proteins have also experienced multiple bands and supposed it as a normal issue [31, 32].

The sera from mice immunized with GST-PDHB-P80 displayed appropriate reactivity against blotted recombinant GST-PDHB-P80 protein. In order to verify immunogenicity of PDHB-P80, we have examined the reactivity of the sera of vaccinated mice with inactivated bacteria vaccine against blotted GST-PDHB-P80. Since this vaccine contains whole cell proteins including PDHB and P80, the observed reactivity refers to the immune reactivity of PDHB-P80. Our findings are consistent with the Sun et al. (2014) and Qi et al. (2018) that demonstrated the immunogenicity of recombinant PDHB protein of *Mycoplasma bovis* and *Mycoplasma gallisepticum* respectively [19, 26]. The results also support the data obtained by Tola et al. (2001) that indicated the immunogenicity of 37-kDa fragment of P80 protein and agree with Fusco et al. that demonstrated the potential of this protein fragment as coating antigen to develop recombinant ELISA for detection of CA [13, 14]. Regarding the promising findings presented in this paper, remaining work on the reaction of the sera from infected goat or sheep with recombinant PDHB-P80 appears fully justified.

In conclusion, we constructed the innovative recombinant protein PDHB-P80 of *Mycoplasma agalactiae* and successfully expressed and purified this chimeric protein. Confirming in silico analysis, experimental immunogenicity evaluations represented appropriate serodiagnostic properties for our proposed novel marker. It can be applied as coating antigen in recombinant ELISA for an effective and rapid diagnosis of both diseased and asymptomatic carriers in CA eradication programs.

## Methods

### Ethics approval and consent to participate

This study was approved by the Animal Ethics Committee (AECs) of School of Veterinary Medicine, Shiraz University (permit: 96GCU2M163973) and all the animal experiments were performed with our institutional guidelines and regulations (dated 20 September 2013) and ARRIVE guidelines for reporting animal research as much as possible (https://arriveguidelines.org/).

### Bioinformatics analysis

The *pdhb-p80* fusion gene was constructed as described previously [30]. Briefly, full-length of *pdhb* gene (accession number CBH40332.1, 984 bp) and antigenic region of *p80* gene (accession number X95628.2, 459 bp) were fused by human middle hinge IgG4 linker (CPSCP) and synthetized after some modifications and optimizing the sequence for expression in *E. coli*. We employed several bioinformatics approaches to predict the physicochemical and antigenic properties of PDHB-P80 fusion protein.

#### Secondary structure prediction

To determine the secondary structure of PDHB-P80 fusion protein, the self-optimized prediction method (SOPMA) was applied by using the available online tools at the following link, (http://npsapbil.ibcp.fr/cgi-bin/npsa_automat.pl?page=/NPSA/ npsa_sopma.html). The similarity threshold and window width were set to 8 and 17, respectively and the percentages of four conformations (α helices, extended strand, β turns and random coils) of the protein were estimated [33].

#### Solubility

Using Pro-Sol online server, we compared the solubility of PDHB-P80 protein with the appropriate solubility of the reference based on amino acid sequence (http://protein-sol.man chester.ac.uk) [34, 35].

#### Hydrophobic regions

The protein-sol patches software was used to predict the ratio of polar to non-polar regions of the fusion protein PDHB-P80 based on the PDB file of sequence generated by I-TASSER [34, 35].

#### B-cell epitope prediction

ElliPro tool provided the conformational B-cell epitopes based on the three-dimensional structure of the protein. PDB file of the protein generated by I-TASSER was used as input and the epitopes with the highest scores were estimated [36].

### Experiments

#### Cloning procedure

The designed fusion gene was synthesized in pMAT cloning vector by Thermo Fisher Scientific Company (U.S.A). The synthetized plasmid pMAT- *pdhb-p80* was transformed into *E. coli* BL21 strain through electroporation and after amplification, plasmid extraction was performed. The pGEX-4 T-1 plasmid was used as an expression vector and were digested along with pMAT-PDHB-P80 using the same restriction enzymes *Bam* HI and *Sal* I (Invitrogen Anza™, Thermo Fisher Scientific, U.S.A). Afterwards, the digestion products were electrophoresed on a 0.75% agarose and extracted by QIAquick Gel Extraction kit (QIAGEN, Germany). Using Anza^TM^T4 DNA ligase master mix (Thermo Fisher Scientific, U.S.A), the purified digested vector and insert were ligated with a ratio of 1 to 3. The recombinant plasmid pGEX-*pdhb-p80* transformed into the competent cells of *E. coli* BL21 via electroporation. After that, the transformed cells incubated at 37 °C for one hour and then plated on 2YT agar containing 100 μg/ml of ampicillin for overnight. To verify the accuracy of cloning procedure, we performed colony PCR by pGEX-4 T-1 specific primers, double digestion analysis on extracted plasmid and Sanger sequencing on obtained colonies [37].

#### Protein expression and cell lysis

To express the recombinant protein, the transformants of *E. coli* including of *pdhb-p80* were cultured in 50 ml 2YT broth at 37 °C overnight in a shaking incubator at 180 rpm. The overnight culture was transferred to one liter of 2YT broth supplemented with 100 µg/ml ampicillin and 0.1% D-Glucose incubated at 37ºC with shaking at 180 rpm. One mM Isopropyl-*β*-D-thiogalactopyranoside (IPTG) was added to the culture at the optical density (OD)_600_ of 0.7. The incubation performed at 25 °C under agitation for four more hours. The cells were harvested by centrifuge of culture fluid at 5000 rpm and 4 °C for 10 min and the pellet was resuspended in phosphate buffered saline (PBS), 1% Triton X-100 (stock solution 20X), 1 mg/ml DNase I, 10 mM MgCl_2_ respectively. The cell suspensions were sonicated in short pulses for ten times then centrifuged at 14000 rpm at 4 °C for 20 min. The supernatant was filtered using 45 µm syringe filter and stored at 4 °C as the cell lysate for next steps [38].

#### Purification of GST-PDHB-P80

The cell lysate was flowed through the immobilized glutathione Sepharose column, thus, the GST tagged protein GST-PDHB-P80 binds to the ligand. The column was washed by three volume of GST-column buffer contained 20 mM Tris–HCl, 150 mM NaCl and 1 mM DTT. The washed column incubated with the GST-elution buffer contained GST-column buffer and 20 mM reduced glutathione for one hour and the purified GST-PDHB-P80 was collected afterwards. Sodium dodecyl polyacrylamide gel electrophoresis (SDS-PAGE) and Western blots analysis using Anti-GST antibody were performed to verify the expression and purification of GST-PDHB-P80. Briefly, resolved proteins on 10% polyacrylamide gel were transferred to nitrocellulose membrane using a Trans-Blot semidry apparatus (Bio-Rad, U.S.A). After the blocking step using 10% bovine serum albumen (BSA) in PBS for overnight, the membrane was washed and incubated in goat Anti-GST antibody in a ratio of 1:1000 for two hours under rotation. After washing, the incubation with 1:200 Anti*-*Goat HRP secondary antibody was performed for two hours and protein bands were visualized using diaminobenzidine (DAB) [38].

#### Immunization procedure

In order to evaluate the immune reactivity of the constructed recombinant protein, 30 female BALB/c mice aged 6–8 weeks (10 animals per each group) obtained from Razi Vaccine and Serum Research Institute, Shiraz, Iran were immunized as follows. Group 1, were injected with PBS for 5 weeks as negative control, group 2, were administered with 200 μl inactivated agalactiae vaccine (contained bacterial suspension 2 × 10^9^ CFU/ml, Horse serum, Saponin, Formaldehyde) (Razi Vaccine and Serum Research Institute, Shiraz, Iran) for two times at 2-week intervals and group 3, immunized with 60 μg recombinant fusion protein GST-PDHB-P80 five times at 2-week intervals. In groups 1 and 3, GST-PDHB-P80 and PBS were emulsified with the same volume of complete Freund’s adjuvant in the first immunization and with incomplete Freund’s adjuvant (Razi Vaccine and Serum Research Institute, Iran) in the following immunizations in a total volume of 200 μl per mouse. After bleeding, we used 1:100 diluted sera raised from the treated animals as primary antibody in Western blot analysis on the blotted GST-PDHB-P80. Goat Anti-Mouse IgG (H L)-HRP antibody (Bio-Rad, U.S.A) was applied at a ratio of 1 to 1000 as secondary antibody. The incubation with sera and secondary antibody performed at room temperature for two hours.

## Supplementary Information

**Additional file 1:** **Fig. 1S. **PDHB-P80 sequence. From nucleotide 1 to 1015 is PDHB sequence and 1027 tothe end is P80 antigenic region. **Fig. 2S.** The hydrophobicity of whole P80 sequence estimated by BioEdit Sequence Alignment Editor. The more antigenic (hydrophilic) regions of P80(from amino acid 373 to 524) were selected to construct the fusion protein. **Fig. 3S.** Separated solubility analysis of PDHB and P80 using Pro-Sol online server. **Fig. 4S.** Secondary structure of PDHB protein estimated by SOPMA online server. **Fig. 5S.** Secondary structure of P80 protein estimated by SOPMA online server.

## Data Availability

The datasets analyzed during this study are available from the corresponding author on reasonable request. The datasets analyzed during the current study are available in the NCBI repository, [Pyruvate dehydrogenase E1 component, β subunit protein accession number: CBH40332.1. (https://www.ncbi.nlm.nih.gov/protein/CBH40332.1/) and *p80* accession number: X95628.2 (https://www.ncbi.nlm.nih.gov/nuccore/X95628].
