# Supplementary material for: A novel fusion protein candidate for the serodiagnosis of Mycoplasma agalactiae infection
Source: BMC Vet Res. 2022 Dec 29;18:456. doi: 10.1186/s12917-022-03558-0 (PMC9798644; doi:10.1186/s12917-022-03558-0)
Supplement: Supplementary file 1 — Additional file 1: Fig. 1S. PDHB-P80 sequence. From nucleotide 1 to 1015 is PDHB sequence and 1027 tothe end is P80 antigenic region. Fig. 2S. The hydrophobicity of whole P80 sequence estimated by BioEdit Sequence Alignment Editor. The more antigenic (hydrophilic) regions of P80(from amino acid 373 to 524) were selected to construct the fusion protein. Fig. 3S. Separated solubility analysis of PDHB and P80 using Pro-Sol online server. Fig. 4S. Secondary structure of PDHB protein estimated by SOPMA online server. Fig. 5S. Secondary structure of P80 protein estimated by SOPMA online server. [file 12917_2022_3558_MOESM1_ESM.docx]

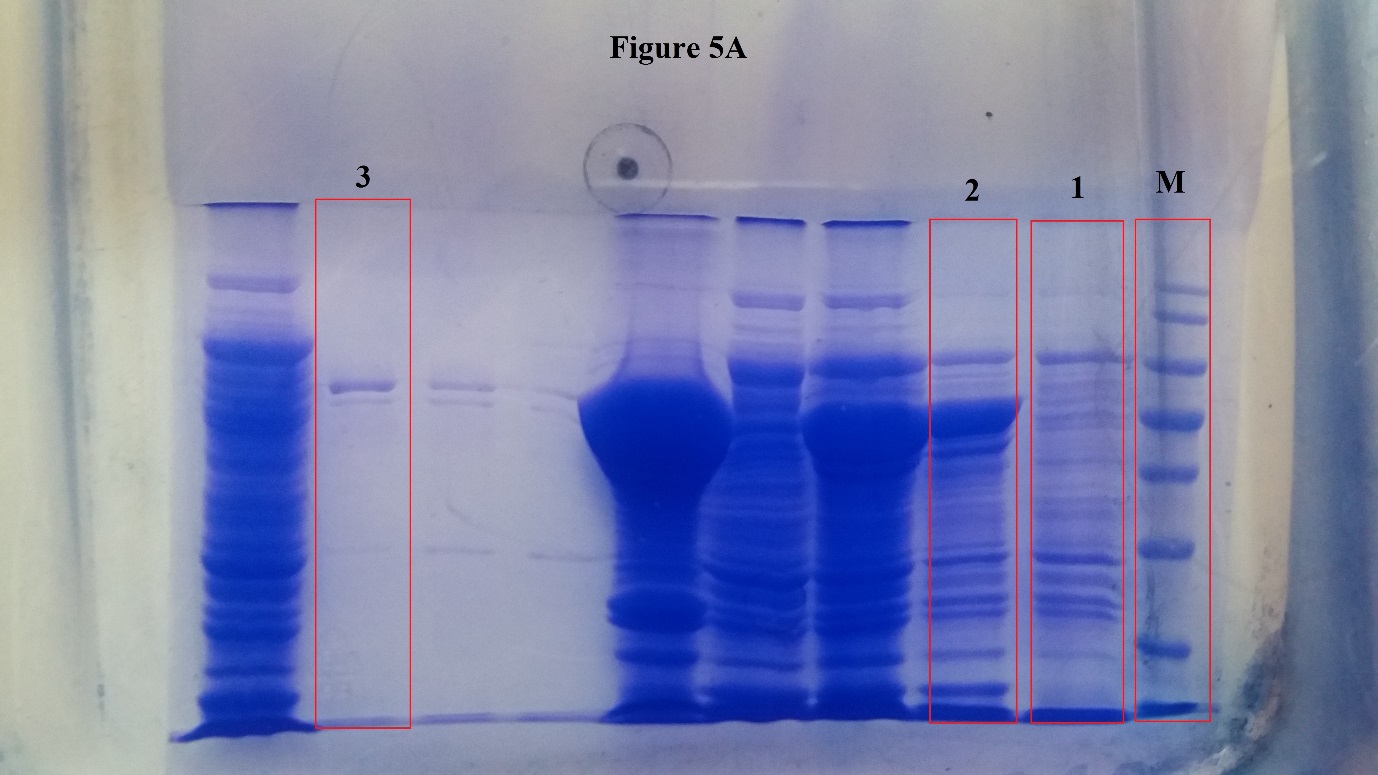


**Fig 5 A**

**
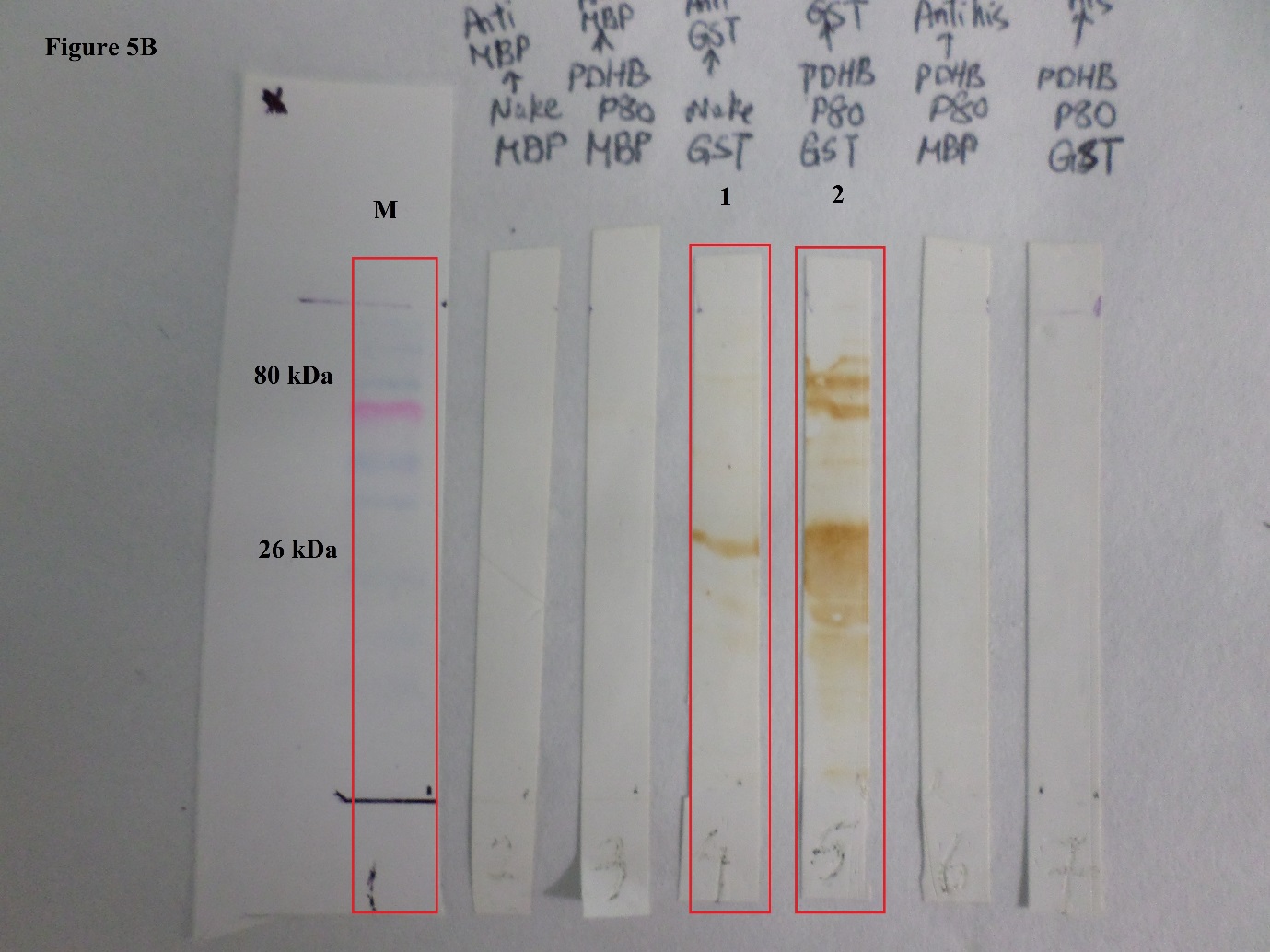
**

**Fig 5 B**

**
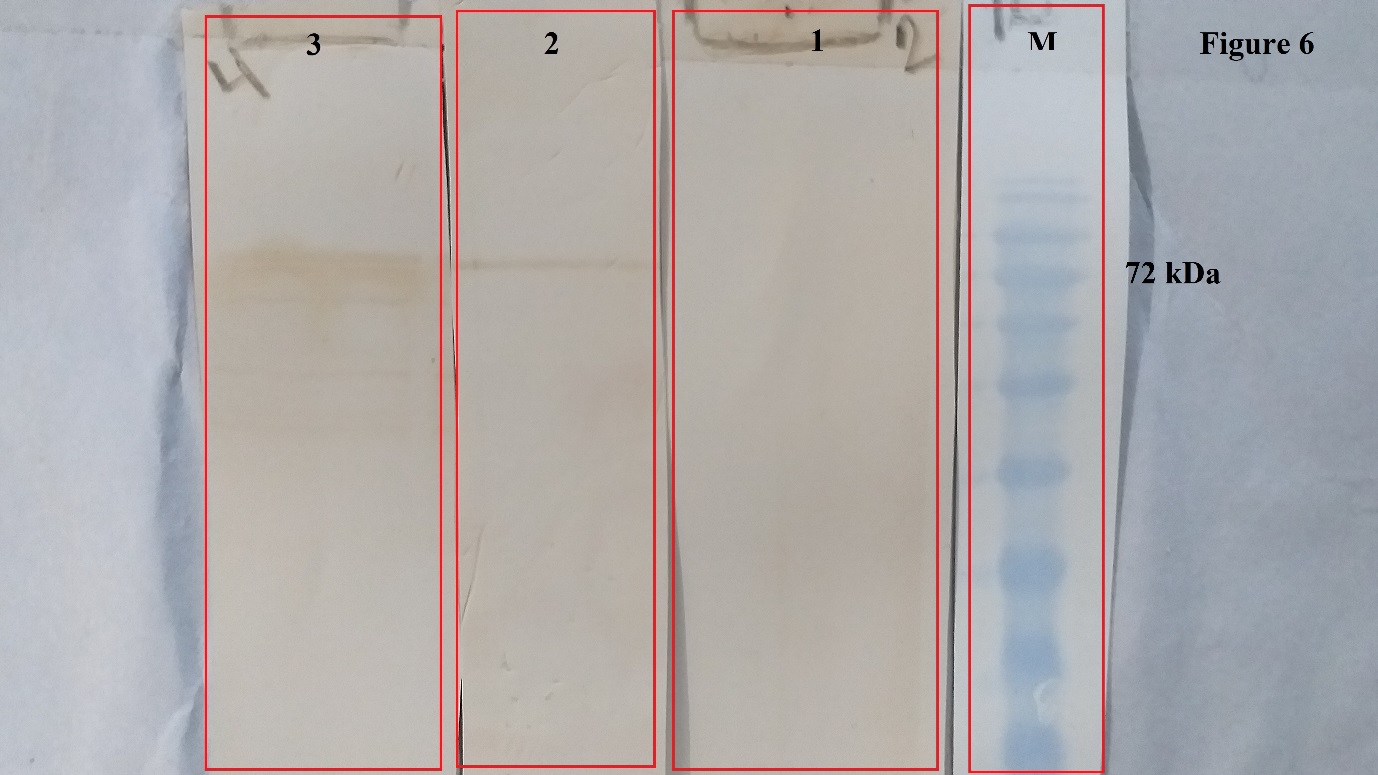
**

**Fig 6**


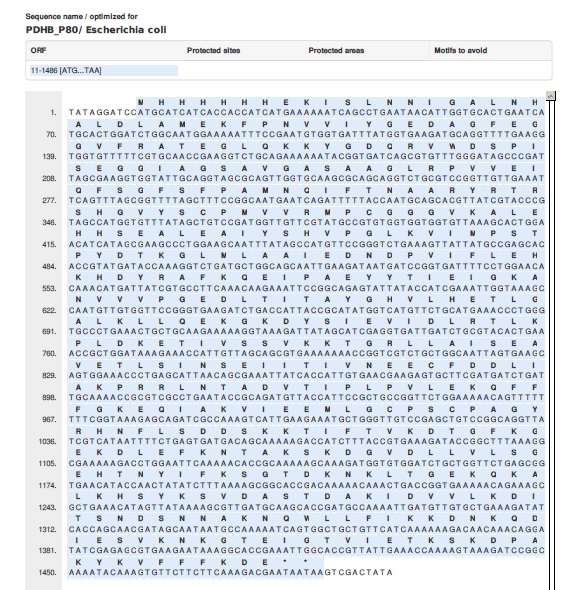


**Fig 1S.** PDHB-P80 sequence. From nucleotide 1 to 1015 is PDHB sequence and 1027 to the end is P80 antigenic region.


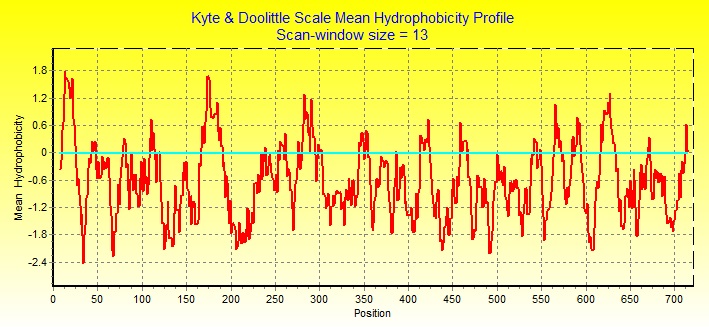


**Fig 2S.** The hydrophobicity of whole P80 sequence estimated by BioEdit Sequence Alignment Editor. The more antigenic (hydrophilic) regions of P80 (from amino acid 373 to 524) were selected to construct the fusion protein.


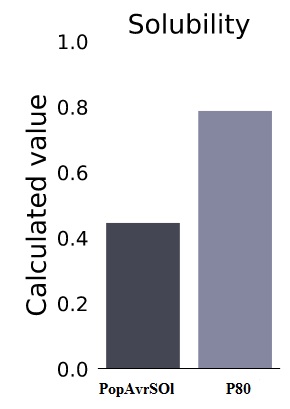

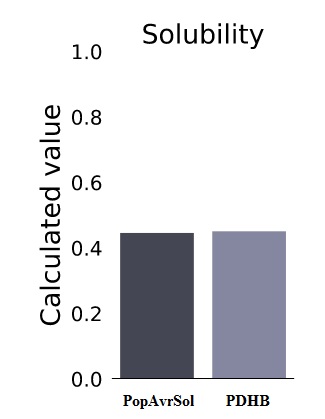


**Fig 3S.** Separated solubility analysis of PDHB and P80 using Pro-Sol online server.


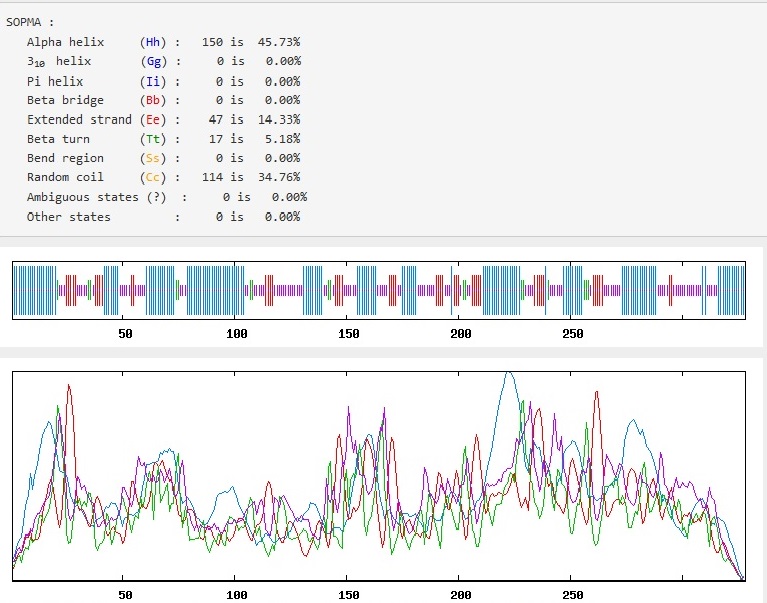


**Fig 4S.** Secondary structure of PDHB protein estimated by SOPMA online server.


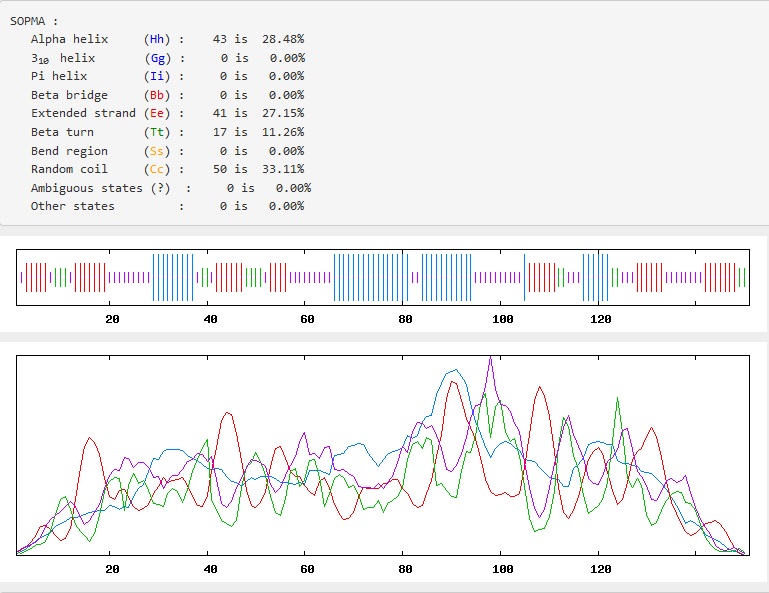


**Fig 5S.** Secondary structure of P80 protein estimated by SOPMA online server.
